# Automated machine learning for differentiation of hepatocellular carcinoma from intrahepatic cholangiocarcinoma on multiphasic MRI

**DOI:** 10.1038/s41598-022-11997-w

**Published:** 2022-05-13

**Authors:** Rong Hu, Huizhou Li, Hannah Horng, Nicole M. Thomasian, Zhicheng Jiao, Chengzhang Zhu, Beiji Zou, Harrison X. Bai

**Affiliations:** 1 Department of Radiology, Xiangya Hospital, Central South University, Changsha, China; 2 Department of Radiology, The Second Xiangya Hospital, Central South University, Changsha, China; 3 Department of Bioengineering, University of Pennsylvania, Philadelphia, PA USA; 4 Warren Alpert Medical School of Brown University, Providence, RI USA; 5 School of Computer Science and Engineering, Central South University, Changsha, China; 6 Department of Radiology and Radiological Sciences, Johns Hopkins University School of Medicine, 601 N Caroline St, Baltimore, MD 21205 USA

**Keywords:** Cancer imaging, Machine learning

## Abstract

With modern management of primary liver cancer shifting towards non-invasive diagnostics, accurate tumor classification on medical imaging is increasingly critical for disease surveillance and appropriate targeting of therapy. Recent advancements in machine learning raise the possibility of automated tools that can accelerate workflow, enhance performance, and increase the accessibility of artificial intelligence to clinical researchers. We explore the use of an automated Tree-Based Optimization Tool that leverages a genetic programming algorithm for differentiation of the two common primary liver cancers on multiphasic MRI. Manual and automated analyses were performed to select an optimal machine learning model, with an accuracy of 73–75% (95% CI 0.59–0.85), sensitivity of 70–75% (95% CI 0.48–0.89), and specificity of 71–79% (95% CI 0.52–0.90) on manual optimization, and an accuracy of 73–75% (95% CI 0.59–0.85), sensitivity of 65–75% (95% CI 0.43–0.89) and specificity of 75–79% (95% CI 0.56–0.90) for automated machine learning. We found that automated machine learning performance was similar to that of manual optimization, and it could classify hepatocellular carcinoma and intrahepatic cholangiocarcinoma with an sensitivity and specificity comparable to that of radiologists. However, automated machine learning performance was poor on a subset of scans that met LI-RADS criteria for LR-M. Exploration of additional feature selection and classifier methods with automated machine learning to improve performance on LR-M cases as well as prospective validation in the clinical setting are needed prior to implementation.

## Introduction

Primary liver malignancy is a leading cause of cancer-related mortality worldwide, and its incidence is on the rise^1,2^. Together, hepatocellular carcinoma (HCC) and intrahepatic cholangiocarcinoma (ICC) comprise the vast majority of cases^3^. HCC is a solid liver tumor resulting from aberrant proliferation of the liver parenchyma, whereas the pathogenesis of ICC involves the dysregulation of epithelioid cells of the biliary tract. These malignancies can be distinguished not only by origin but also in terms of their clinical progression. Differences in individual tumor burden notwithstanding, ICC tends to present more aggressively and usually portends a worse prognosis when compared to HCC^4–6^. Clinical interventions for both conditions are guided by diagnosis, so proper tumor classification is essential for appropriately targeting therapy.

Non-invasive imaging with ultrasound, computed tomography (CT), contrast enhanced ultrasound, and magnetic resonance imaging (MRI) are mainstays in the clinical management primary liver carcinoma. In fact, with recent advancements in medical imaging resolution, the American Association for the Study of Liver Disease practice guidelines recommend HCC diagnosis on imaging findings alone, with tissue biopsy only indicated in a select number of indeterminate cases^7,8^. Among these imaging techniques, MRI has emerged as a leading visualization modality in primary liver cancer diagnostics, as it can overcome limitations in ultrasound and CT in terms of resolution and radiation exposure, respectively^9,10^. The literature also highlights the potential to leverage MRI for disease surveillance, as it is associated with higher sensitivity for detecting HCC than screening with ultrasonography and CT^10,11^.

To date, the diagnosis of HCC on imaging is based on the identification of vascular features, such as hypervascularity in the arterial phase coupled with wash-out in the portal-venous or the delayed phase, which are typical of overt HCC^12,13^. However, differences in the mass-forming subtype of intrahepatic cholangiocarcinoma (mICC), which make up a majority of ICC cases, may be more subtle^14,15^. This radiographic mimic can appear similar to poorly differentiated, hypovascular HCC variants^16,17^. Alternatively, small mICCs (< 3 cm in diameter) can be read as hypervascular on the arterial phase, which can be misinterpreted as HCC^18^. Diagnosis is further complicated by the fact that elevations in biomarkers of HCC can also be seen in ICC^19,20^.

The liver imaging reporting and data system (LI-RADS) was developed to support radiologists’ diagnostic evaluations by providing a scale reflecting the probability of HCC. The labels LR-1 to LR-5 correspond to the likelihood of hepatocellular carcinoma (HCC). LI-RADS also provides a LR-M category for lesions that are definitely or probably malignant but that are not specific to HCC. The features of non-HCC malignancy (LR-M) defined by LI-RADS are most closely associated with the imaging appearance of ICC, as this is the most common non-hepatocellular primary liver tumor. Most typical appearing ICCs can be reliably identified as LR-M with LI-RADS, however atypical variants are more likely to result in a false positive LR-5/5v classification of HCC^21^. In addition, studies assessing interrater agreement on LI-RADS categories found “fair” to “substantial” concordance on MRI, reflecting some degree of ambiguity in this framework intended to standardize reporting^22^.

To bridge this gap, we envision a role for machine learning in the diagnosis of primary liver cancer as an adjunct to conventional radiologist imaging evaluation^23–26^. Radiomics is a sub-field of machine learning that refers to the conversion of diagnostic images to mineable pixel data via high-throughput extraction of quantitative features^27^. Imaging features linked to pathology-proven diagnoses are then used to train a classifier that can be used in support of clinical decision making. Quantification of image analysis with radiomics may confer an advantage over conventional radiology through standardization and, in some cases, inclusion of imaging features that may not be perceptible to the human eye. The traditional radiomics workflow consists of multiple steps including image acquisition, feature extraction, feature selection, and model selection^28^. Prior studies using machine learning-based radiomics for differentiation of HCC from ICC have primarily utilized CT imaging^29,30^. While these efforts are a laudable first step in improving diagnostics, multiphasic MRI can provide additional tumor detail that may translate to improved classifier performance^31^.

Recent advancements in radiomics also raise the possibility of implementing automated machine learning (AutoML) tools to accelerate workflow^32^. Automated machine learning applications in radiomics have demonstrated non-inferior performance in terms of accuracy and computing efficiency when compared to conventional methods^33,34^. The primary utility of AutoML is as an alternative to manually calculating optimal feature transformation and classifier combinations. Manual optimization in radiomics also requires computational expertise that may preclude its use by mainstream clinical researchers. Thus, AutoML has the potential to enhance both the accessibility and scalability of machine learning pipeline engineering.

The Tree-Based Pipeline Optimization Tool (TPOT) is a one such AutoML tool in Python that uses a genetic search algorithm to automate the feature extraction, feature selection, and model selection steps of the radiomics workflow to maximize classification accuracy^28,35^. The output of the TPOT algorithm consist of several operators indicating the optimal machine learning pipeline, which specify the feature transformer, classifier, and parameter optimizer. TPOT is distinguished from other automated machine learning pipeline optimization tools like auto-sklearn^36^, auto_ml^37^, H_2_O automl^38^,auto-tune models^39^, and ML box^40^ by the use of its genetic programming, which allows it to identify more unique pipelines. It can export executable Python code implementing the best pipeline allowing the data scientist to further modify the generated model. TPOT software has already been applied to several domains including genomics, placental MRI, brain MRI, and metabolite/lipoprotein profiling^41–43^. Within the field of hepatology, TPOT has been explored for use in staging and prediction of hepatocellular tumor response to transcatheter arterial chemoembolization^44,45^. In this study, we hypothesize that our AutoML model selected with TPOT will have a performance comparable to that of radiologists on differentiation of HCC from ICC on multiphasic MRI.

## Materials and methods

### Patient cohorts

This study was Health Insurance Portability and Accountability Act (HIPAA) compliant and approved by the Institutional Review Board (IRB) at all 3 institutions (Second Xiangya Hospital, Hospital of the University of Pennsylvania, and Rhode Island Hospital). The requirement for informed consent was waived by IRB of Second Xiangya Hospital, Hospital of the University of Pennsylvania, and Rhode Island Hospital. All methods were performed in accordance with the relevant guidelines and regulations. Retrospective acquisition of pathology records for HCC and ICC from 2008 to 2018 was obtained via electronic medical record search. Only patients with diagnosis confirmed by pathology were included in the study. All patients had MRI imaging of the abdomen prior to operation or biopsy, with arterial and portal venous phase images included in the acquisition protocol. MR scanning parameters for the Second Xiangya Hospital (SXY) and the Hospital of the University of Pennsylvania (HUP) cohorts on T1 with contrast (T1C) and T2-weighted (T2W) sequences are presented in Supplementary Fig. S1. Given the technical difficulty in accurately segmenting smaller tumors, 48 lesions with a size of less than 2 cm in all dimensions were excluded. Patients that met the following criteria were also excluded from analysis: (1) liver tumors with mixed-cell carcinoma; (2) time from MRI examination to pathological diagnosis of greater than one year; (3) images with incomplete or inappropriate imaging protocols; and (4) images with motion or other artifact impeding interpretability. The final cohort included 282 patients with HCC and 207 patients with ICC, all with lesions measuring at least 2 cm in all dimensions with clearly demarcated borders. There were 24 cases (HCC vs ICC = 5:19) that satisfy LR-M criteria in the test set.

### Tumor segmentation

**Figure 1:**
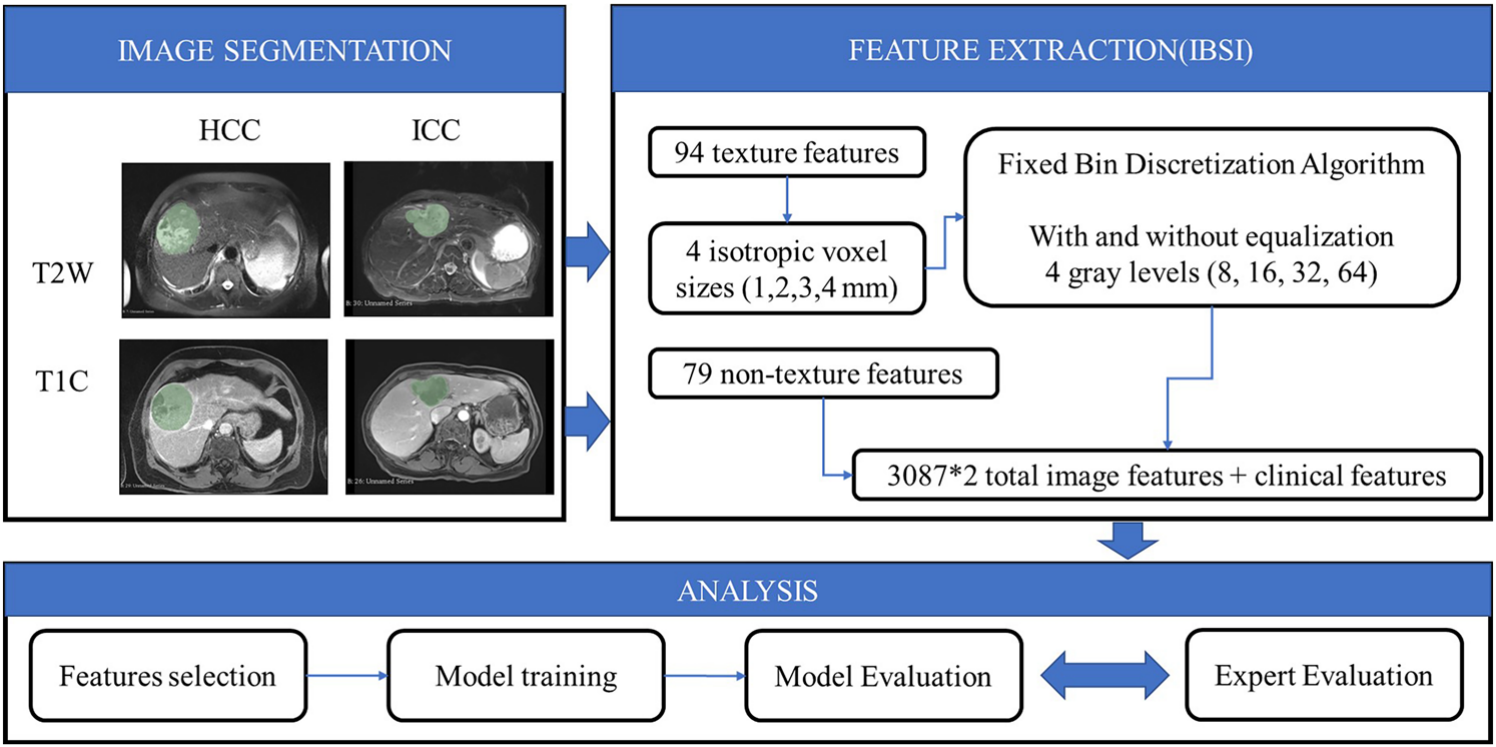
Workflow for radiomics feature extraction. Physicians segmented the tumor on MRI scans. The segmented region was then used to extract radiomics features of the tumor, which were used to classify tumors as HCC or ICC based on pathologic diagnosis.

Manual segmentation of hepatic tumors was performed by a radiologist using 3D Slicer software^46^. The boundary of the tumor was carefully drawn by the radiologist on each axial slice of the T1C and T2W sequences. Care was taken to include all voxels of the entire tumor, as demonstrated in Fig. 1. Segmentation of the test set was performed twice and by two different radiologists of similar expertise level to ensure reproducibility and consistency of the results. Kappa scores for inter-rater variability between radiologists are depicted in Table 1. Fleiss Kappa among all experts were 0.52, which corresponds to moderate interrater variability^47^.

**Table 1:** Kappa scores for inter-rater variability among radiologists on test set.

	Radiologist 1	Radiologist 2	Radiologist 3	Radiologist 4	Outcome
Radiologist 1	1.00	0.28	0.44	0.53	0.53
Radiologist 2	0.28	1.00	0.65	0.57	0.66
Radiologist 3	0.44	0.65	1.00	0.65	0.74
Radiologist 4	0.53	0.57	0.65	1.00	0.74

### Feature extraction

514 patients were randomly divided into independent training (HCC vs ICC = 198:146), validation (HCC vs ICC = 56:41) and test (HCC vs ICC = 28:20) sets. There were 24 cases (HCC vs ICC = 5:19) that satisfied LR-M criteria in the test set^48^. Radiomics features were extracted in accordance with Image Biomarker Standardization Initiative (IBSI) guidelines from each patient MRI scan, including both T1C and T2W sequences (Fig. 1)^49,50^. There were 79 non-texture and 94 texture features. Non-texture features including morphological features, local intensity features, intensity-based statistical features, intensity histogram features and intensity-volume histogram features were included in the analysis. Texture feature computation was performed by extracting gray level co-occurrence-based features, gray level run length-based features, gray level distance zone-based features, neighborhood gray tone difference-based features and neighboring gray level dependence-based features^51–53^. Each feature was calculated 32 times using 4 (1, 2, 3, 4 mm) isotropic voxel size with and without equalization of a fixed bin number discretization algorithm using 4 (8, 16, 32, 64) gray levels. There were 3087 (79 + 94 × (4 × 2 × 4)) features computed for each sequence. Clinical variables of age and sex were also included in the study, yielding a total of 6176 (3087 × 2 + 2) included in the feature set. Features with “NaN” in all cases were removed. A total of 6130 features were used in the subsequent analysis.

### Feature selection and classification

Features were first normalized using Min–Max Scaling, and different thresholds of variance-based feature selection (VBFS) were used in combination with Support Vector Machine (SVM), Random Forest (RF), and Multilayer Perceptron (MLP), XGBoost, AdaBoost, Extra Trees (ET), Logistic Regression (LR), Gradient Boosting (GB) classifiers on the training set. First, each combination of threshold and classifier were evaluated using ten-fold cross-validation on training set, and mean area under the curve (ROC AUC) (cv-scores) were recorded. Next, the threshold of VBFS that achieved the highest mean ROC AUC on ten-fold cross-validation process was selected for each classifier. Then, the combinations of optimal threshold and classifiers were trained on the whole training set and subsequently applied on validation set. The combination that achieved the highest ROC AUC on validation set was evaluated on the test set.

**Figure 2:**
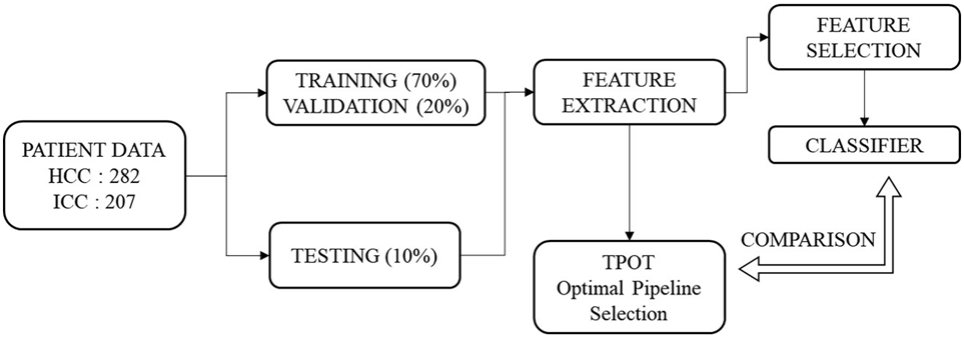
Workflow for classification analysis.

For the AutoML approach, the TPOT classifier was run 10 times across 10 generations and with population size of 20. In each run, TPOT generated 10 model pipelines randomly to form the first generation, which were subsequently trained and evaluated. The top performing model pipelines were conserved to form 10% of the new population. Next, three model pipelines in the existing population were eliminated in “natural selection” style approach employed by the genetic search algorithm, whereby the simplest pipeline that achieved the best performance was reproduced in the new population. This selection process continued until the remaining 90% of the new population was generated. Next, a one-point crossover was performed where the algorithms splits and swaps the contents of two model pipelines selected from the existing population random, with a mutation operation performed on the remaining pipelines. This process was repeated for each generation. Finally, the single best-performing pipeline ever discovered with the genetic search algorithm was exported by TPOT. We ran the TPOT default configuration and TPOT Light configuration, as they specified different operators. The pipeline exported by TPOT with the highest ROC AUC on the validation set was selected, and the accuracy, sensitivity, specificity, ROC AUC, area under the precision recall curve (PR AUC), and Kappa score were calculated on the test set. All models were run on a Windows 10, Intel(R) Core(TM) i7-8700 CPU @3.2 GHz 64 GB. The workflow of manual and automatic classification was illustrated in Fig. 2.

### Radiologist evaluation

Four radiologists (Y.Y.C, Y.J.Z, D.L., and W.J.) with 3, 4, 4, and 8 years, respectively, of experience reading abdominal MRI were blinded to the histopathological data. The radiologists classified unsegmented MRI images of test set liver lesions as HCC or ICC. To quantify performance differences between the machine learning models and radiologists, a p-values and 95% confidence intervals (CI) were calculated using a binomial test.

## Results

### Manual optimization

Model performance on the training set at varying thresholds of selected features is shown in Supplementary Fig. S2, which indicates XGBoost and LR were the top 2 most robust classifiers. The optimal VBFS threshold for each classifier and their performance on validation set is presented in Supplementary Table S1. The VBFS (threshold = 0.031) and Logistic Regression (LR) (VBFS + LR) achieved the best performance on the validation set with manual optimization. The VBFS + LR model achieved a ROC AUC of 0.89, accuracy of 84% (95% CI 0.75–0.90), sensitivity of 76% (95% CI 0.61–0.87) and specificity of 89% (95% CI 0.78–0.95) on validation set (See Supplementary Table S1). On the test set, the VBFS + LR had a ROC AUC of 0.79–0.80, accuracy of 73–75% (95% CIs 0.59–0.85), sensitivity of 70–75% (95% CIs 0.48–0.89), and specificity of 71–79% (95% CI 0.52–0.90) (See Table 2). It took approximately 20 min to find an optimal threshold and to train each classifier by hand (20 min × 3 models = 60 min total).

**Table 2:** Test set performance accuracy and 95% confidence intervals of hand- versus automated-optimized models for 1st and 2nd segmentation with radiologist comparison.

	ROC AUC	PR AUC	Accuracy	P value	Sensitivity	P value	Specificity	P value	Kappa
**Segmentation 1**									
Radiomics pipeline (VBFS + LR)	0.80	0.81	0.73 (0.59–0.84)	0.05	0.75 (0.53–0.89)	0.58	0.71 (0.52–0.85)	0.007	0.45
TPOT	0.76	0.76	0.73 (0.59–0.84)	0.05	0.65 (0.43–0.82)	0.10	0.79 (0.61–0.90)	0.25	0.44
**Segmentation 2**									
Radiomics pipeline (VBFS + LR)	0.79	0.80	0.75 (0.61–0.85)	0.11	0.70 (0.48–0.86)	0.26	0.79 (0.61–0.90)	0.25	0.49
TPOT	0.79	0.77	0.75 (0.61–0.85)	0.11	0.75 (0.53–0.89)	0.58	0.75 (0.56–0.88)	0.08	0.49
Radiologist 1	NA	NA	0.77 (0.63–0.87)	0.11	0.75 (0.53–0.89)	0.58	0.79 (0.61–0.90)	0.25	0.53
Radiologist 2	NA	NA	0.83 (0.70–0.91)	0.56	0.80 (0.58–0.93)	1.00	0.86 (0.68–0.95)	0.78	0.66
Radiologist 3	NA	NA	0.88 (0.76–0.95)	0.69	0.80 (0.58–0.93)	1.00	0.93 (0.76–0.99)	0.57	0.74
Radiologist 4	NA	NA	0.88 (0.76–0.95)	0.69	0.85 (0.63–0.96)	0.78	0.89 (0.72–0.97)	0.79	0.74
Mean radiologist	NA	NA	0.84 (0.71–0.92)	1.00	0.80 (0.58–0.93)	1.00	0.87 (0.69–0.96)	1.00	NA

### Automated optimization

The performance of automated machine learning with TPOT on validation set are presented in Supplementary Table S2 (TPOT Light configuration) and Supplementary Table S3 (TPOT default configuration). The AutoML Random Forest (RF) classifier (exported by No.9 TPOT) achieved a ROC AUC of 0.83 (higher than the best Logistic Regression exported by TPOT Light) on validation set with an accuracy of 77% (95% CI 0.68–0.84), sensitivity of 0.76% (95% CI 0.61–0.87), and specificity of 79% (95% CI 0.66–0.88) (See Supplementary Table S4). On the test set, the AutoML model achieved a ROC AUC of 0.76–0.79, accuracy of 73–75% (95% CI 0.59–0.85), sensitivity of 65–75% (95% CI 0.43–0.89), and specificity of 75–79% (95% CI 0.56–0.90) (See Table 2). Automated optimization with TPOT Light took 14 min per run and TPOT took 6 h per run, in which time the algorithm evaluated 10 models (generations = 10, population_size = 20, config_dict = ‘TPOT light’/None, cv = 10).

### Performance evaluation

Pairwise comparisons of classification accuracy between the manually optimized pipeline (VBFS + LR), automatically optimized pipeline, and radiologists are summarized in Table 2. The manually optimized model achieved comparable accuracy (0.73 vs 0.73, p = 1.00), sensitivity (0.75 vs 0.65, p = 0.31), and specificity (0.71 vs 0.79, p = 0.16) when compared with the TPOT pipeline on the test set. TPOT achieved results comparable to that of radiologists in terms of sensitivity (TPOT: 0.80 vs 0.65, p = 0.10) and specificity (0.87 vs 0.79, p = 0.25).

**Figure 3:**
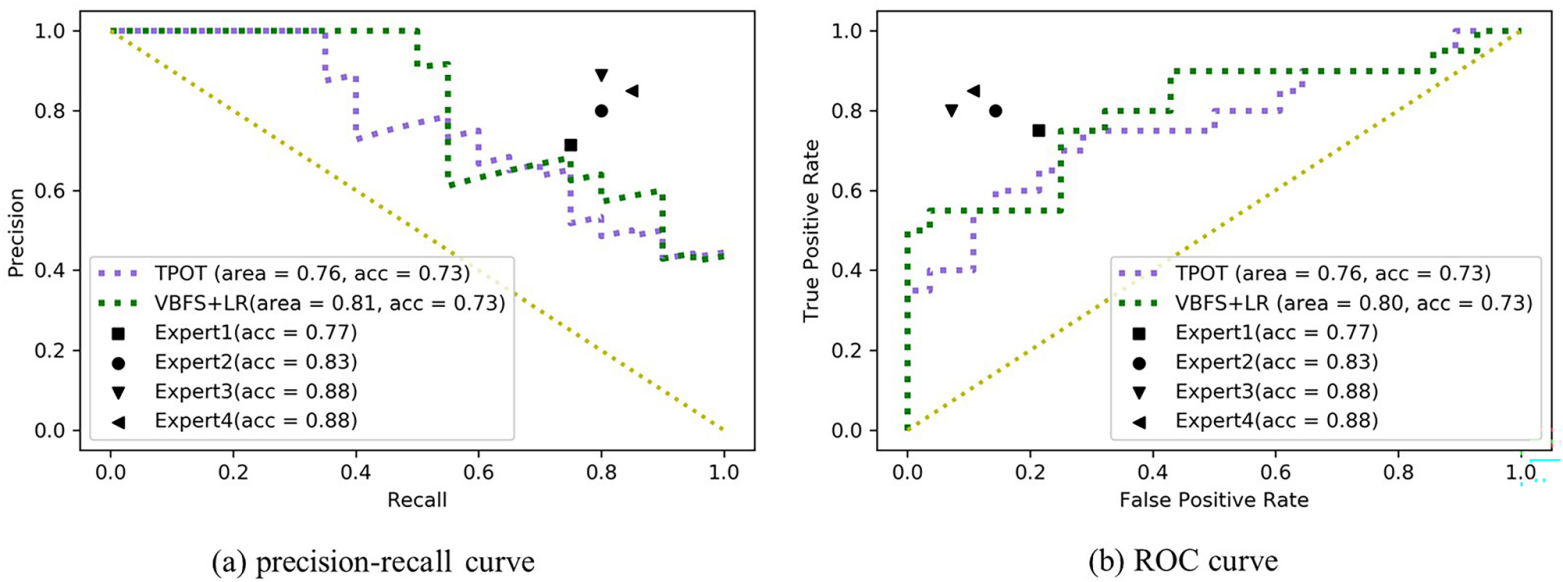
The precision-recall and ROC curves for the VBFS and LR combination, TPOT pipeline, and radiologist evaluations on the testing set.

The test set precision-recall and ROC curves for the optimized pipelines and radiologist evaluations are shown in Fig. 3. To verify the repeatability of the segmentation, images in the test set were segmented by two experts. Our models achieved similar results using these two segmentations in terms of accuracy (VBFS + LR: 0.73 vs 0.75, p = 0.74; TPOT: 0.73 vs 0.75, p = 0.74), sensitivity (VBFS + LR: 0.75 vs 0.70, p = 0.81; TPOT: 0.65 vs 0.75, p = 0.31) and specificity (VBFS + LR: 0.71 vs 0.79, p = 0.16; TPOT: 0.79 vs 0.75 p = 0.83).

For cases in the test set that satisfied the LR-M criteria, manual VBFS + LR achieved a ROC AUC of 0.68, accuracy of 71% (95% CI 0.51–0.85), sensitivity of 79% (95% CI 0.56–0.92), and specificity of 40% (95% CI 0.12–0.77) (See Table 3). The manual VBFS + LR model could distinguish LR-M with similar accuracy (0.71 vs 0.81, p = 0.20) and sensitivity (0.79 vs 0.80, p = 1.00) as radiologists. The automated TPOT model achieved a ROC AUC of 0.60, accuracy of 63% (95% CI 0.43–0.79), sensitivity of 68% (95% CI 0.45–0.85), and specificity of 40% (95% CI 0.12–0.77). The AutoML model distinguished LR-M with a lower accuracy (0.63 vs 0.81, p = 0.03) than that of radiologists.

**Table 3:** Classification accuracy and 95% confidence intervals for machine learning models on the LR-M test set with average radiologist performance comparison on the 1st test-set segmentation.

	ROC AUC	PR AUC	Accuracy	P value	Sensitivity	P value	Specificity	P value	Kappa
Manual	0.68	0.91	0.71 (0.51–0.85)	0.20	0.79 (0.56–0.92)	1.00	0.40 (0.12–0.77)	0.03	0.18
TPOT	0.60	0.89	0.63 (0.43–0.79)	0.03	0.68 (0.45–0.85)	0.08	0.40 (0.12–0.77)	0.03	0.07
Radiologist 1	NA	NA	0.79 (0.59–0.91)	0.44	0.79 (0.56–0.92)	1.00	0.80 (0.36–0.98)	0.56	0.48
Radiologist 2	NA	NA	0.79 (0.59–0.91)	0.44	0.79 (0.56–0.92)	1.00	0.80 (0.36–0.98)	0.56	0.48
Radiologist 3	NA	NA	0.83 (0.63–0.94)	0.80	0.79 (0.56–0.92)	1.00	1.00 (0.51–1.00)	1.00	0.61
Radiologist 4	NA	NA	0.83 (0.63–0.94)	0.80	0.84 (0.61–0.95)	1.00	0.80 (0.36–0.98)	0.56	0.56
Mean radiologist	NA	NA	0.81 (0.61–0.92)	1.00	0.80 (0.57–0.93)	1.00	0.85 (0.40–1.00)	1.00	NA

## Discussion

We developed a series of machine learning feature selection and classifier combinations for the differentiation of ICC from HCC on multiphasic MRI. We also established a novel application of AutoML with TPOT to liver cancer imaging diagnostics. TPOT identified a Random Forest (RF) classifier that closely approximated the performance of the hand optimized VBFS + LR model. Automated machine learning achieved similar sensitivity and specificity to radiologists when assessed on an independent test set. We also evaluated model performance on a dedicated LR-M test set using the LI-RADS criteria, which showed comparable performance accuracy across the hand-optimized pipeline (VBFS + LR) and radiologists. The automated pipeline (RF) did not perform on par with radiologists in terms of performance accuracy for the LR-M subset. Looking at the LR-M test set prediction instances, it appears the AutoML model had the most difficult differentiating HCC cases that met the LR-M criteria as ICC, which is consistent with the results of current literature about limitations in the LI-RADS framework^54,55^.

Our study builds upon prior work demonstrating the utility of CT-based machine learning systems in the classification of primary liver cancer^56^. It also dovetails nicely with past MRI-based radiomics studies that utilize texture analysis to differentiate benign and malignant soft-tissue tumors^57^. To our knowledge, our study is the first in the literature to explore AutoML for the differentiation of primary liver cancers. AutoML can accelerate and simplify the time-intensive model analysis process, which may promote the adoption of machine learning by clinician researchers with a working proficiency in data science or in collaboration with engineering colleagues^58^. It is possible to package AutoML in GUI that can be used by clinicians without background in computer programming. This is important as physician involvement in informatics research is needed to guide the development of clinically meaningful healthcare technologies, particularly in primary liver cancer management where the expertise of hepatologists and radiologists has implications for patient outcomes^59–62^. Looking ahead, there is also a potential to leverage AutoML for other applications in this context, such as to augment liver cancer treatment response evaluation, therapeutic agent selection, and prognostication.

Our study supports the use of the TPOT pipeline as a valuable resource that physicians can use to streamline the machine learning model optimization process. The TPOT platform uses a genetic search algorithm to collapse feature and model selection into a single process that maximizes classification accuracy^33^. Our study showed comparable performance between pipeline selected by TPOT and the manually optimized algorithm (VBFS + LR). In addition, automatic optimization of ten machine learning pipelines with TPOT Light took 14 min and TPOT took 6 h per run (10 models), whereas the manual optimization took 20 min per model.

We acknowledge several limitations in our study design. First, our analysis does not account for the full breadth of primary liver cancers, especially for the lesions ≤ 2 cm. This is very important because the goal of liver surveillance is to identify small sized nodules, as early detection confers a survival benefit. Likewise, as periductal infiltrative and intraductal types of ICC are not routinely biopsied, they were notably absent from the study population and should be considered a limitation to overall generalizability. Although we did our best to balance the patients with HCC and ICC included in the study, there are still differences in baseline characteristics between the two groups across the training, validation, and test sets. Kappa values for the study demonstrate moderate inter-observer agreement (k = 0.52) between most radiologists, and our measurements are similar to others reported in the literature^21,47,63^. Agreement was lower between Radiologists 1 and 2 (k = 0.28), which may be explained by their expertise as junior radiologists. Finally, this study was limited by data availability and would have benefited from larger institutional cohort sizes that would have allowed for use of an external test set. In terms of the automated machine learning analysis, it is worth noting that TPOT performance is more prone to instability when working with large datasets where more run time is needed to arrive at the optimal pipeline. We ran the TPOT ten times to mitigate this limitation with excellent convergence on our selected pipeline (See Supplementary Tables S2–S3: Pipeline 10).

In sum, our study demonstrates a potential role for emerging automated machine learning tools in streamlining the informatics backend workflow, however additional work is needed to improve model performance on LR-M cases. Subsequent testing will evaluate the performance of alternate TPOT evolutions such as TPOT-Pareto and TPOT-DS, Future work will explore techniques for automatic lesion segmentation using the manual labels generated from the study as well as deep learning methods for use in this context. Finally, prospective validation of machine learning model performance in a variety of clinical settings with radiologists using the LI-RADS reporting format is needed prior to implementation.

## Supplementary Information

Supplementary Information.
