# Supplementary material for: Automated machine learning for differentiation of hepatocellular carcinoma from intrahepatic cholangiocarcinoma on multiphasic MRI
Source: Sci Rep. 2022 May 13;12:7924. doi: 10.1038/s41598-022-11997-w (PMC9106680; doi:10.1038/s41598-022-11997-w)
Supplement: Supplementary file 1 — Supplementary Information. [file 41598_2022_11997_MOESM1_ESM.pdf]

Automated machine learning for differentiation of hepatocellular carcinoma from intrahepatic cholangiocarcinoma on multiphasic MRI

Authors: Rong Hu, MD[1*]; Huizhou Li, MD[2*]; Hannah Horng, BS[3]; Nicole M Thomasian, BS[4]; Zhicheng Jiao, PhD[4]; Chengzhang Zhu, PhD [5]; Beiji Zou, PhD [5]; Harrison X. Bai, MD[6†]

Affiliations

[1] Department of Radiology, Xiangya Hospital, Central South University, Changsha, China

[2] Department of Radiology, The Second Xiangya Hospital, Central South University, Changsha, China

[3] Department of Bioengineering, University of Pennsylvania, PA

[4] Warren Alpert Medical School of Brown University, Providence, RI

[5] School of Computer Science and Engineering, Central South University, Changsha, China

[6] Department of Radiology and Radiological Sciences, Johns Hopkins University School of Medicine, Baltimore, MD

*These authors contributed equally and are considered co-first authors

† Corresponding Author: Harrison Bai, Department of Radiology and Radiological Sciences, Johns Hopkins University School of Medicine, 601 N Caroline St, Baltimore, MD, 21205, USA. Email: hbai7@jhu.edu

Supplementary Figure S1 Echo time and Repetition Time for T1C (the first row) and T2W (the second row) images of (a) The Second Xiangya Hospital (SXY) (b) Hospital of the University of Pennsylvania(HUP).

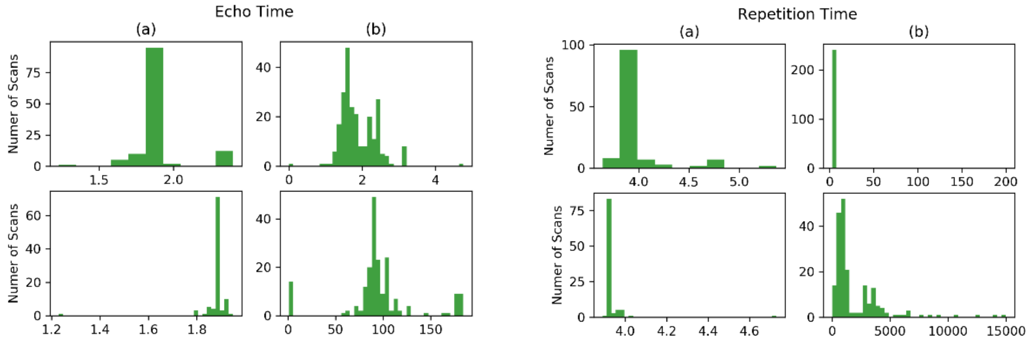

Supplementary Figure S2. Performance of Classifiers on training set using 10 cross-validation with different thresholds of variance-based feature selection applied.

threshold	SVM	MLP	RF	xgboost	Adaboost	ET	LR	GB
0.01	0.774204	0.784863	0.798123	0.817751	0.767791	0.775438	0.803172	0.791893
0.011	0.774157	0.792281	0.801443	0.816768	0.758078	0.787058	0.800392	0.784954
0.012	0.775555	0.767251	0.789577	0.819774	0.757143	0.778038	0.803564	0.78765
0.013	0.774508	0.745912	0.806076	0.813197	0.767806	0.788696	0.806315	0.800148
0.014	0.774841	0.784901	0.802656	0.81634	0.763695	0.773875	0.804546	0.788653
0.015	0.775858	0.5045	0.800775	0.818149	0.748635	0.786188	0.803251	0.802091
0.016	0.775144	0.782335	0.790804	0.816857	0.786639	0.775581	0.804238	0.797001
0.017	0.776192	0.68769	0.790343	0.818643	0.781598	0.785971	0.807321	0.807583
0.018	0.77687	0.788198	0.79394	0.819703	0.767014	0.784579	0.809732	0.792561
0.019	0.777197	0.8059	0.798837	0.817602	0.781706	0.780523	0.80856	0.796308
0.02	0.778922	0.785584	0.789802	0.814494	0.75098	0.788857	0.808965	0.79552
0.021	0.7796	0.780137	0.794489	0.815987	0.766307	0.790754	0.810405	0.80594
0.022	0.778492	0.583011	0.787083	0.814481	0.775871	0.791917	0.809965	0.798262
0.023	0.775713	0.594096	0.799481	0.816856	0.754152	0.785723	0.81059	0.801159
0.024	0.775718	0.796816	0.789311	0.817873	0.791837	0.785759	0.811078	0.803054
0.025	0.774302	0.792094	0.79166	0.820023	0.777926	0.792585	0.819132	0.803571
0.026	0.774986	0.794107	0.791227	0.821373	0.766713	0.780086	0.815858	0.804743
0.027	0.771183	0.79897	0.803648	0.819213	0.774652	0.798237	0.815799	0.80407
0.028	0.768754	0.800112	0.794452	0.819607	0.784249	0.791645	0.822222	0.82291
0.029	0.768761	0.770713	0.782001	0.820059	0.792325	0.792473	0.822833	0.807398
0.03	0.768362	0.797516	0.785445	0.822576	0.789922	0.782443	0.822875	0.808638
0.031	0.767356	0.750786	0.797816	0.819791	0.778108	0.781267	0.828494	0.81454
0.032	0.767719	0.739045	0.794731	0.821148	0.783786	0.768352	0.827916	0.806987
0.033	0.769754	0.579321	0.791528	0.822576	0.787469	0.781865	0.824459	0.813856
0.034	0.7681	0.797292	0.801855	0.823499	0.783783	0.773224	0.826415	0.817487
0.035	0.772694	0.792154	0.80248	0.821576	0.778038	0.788137	0.827713	0.80954
0.036	0.777802	0.795095	0.789998	0.819416	0.832954	0.773167	0.826391	0.815595
0.037	0.780897	0.784678	0.783457	0.814613	0.808658	0.777704	0.823284	0.822783
0.038	0.784075	0.790035	0.786221	0.811595	0.764861	0.776029	0.824142	0.798728
0.039	0.78232	0.804565	0.795021	0.814173	0.791509	0.770092	0.823076	0.814459
0.04	0.779659	0.784678	0.797816	0.817917	0.779837	0.764133	0.823713	0.81966
0.041	0.778261	0.790559	0.784367	0.807216	0.786799	0.781982	0.825802	0.80456
0.042	0.781237	0.798743	0.779255	0.803247	0.765529	0.774313	0.82238	0.814385
0.043	0.779946	0.806107	0.779577	0.807734	0.773441	0.773516	0.820273	0.805694
0.044	0.777857	0.794494	0.780506	0.79467	0.779132	0.777469	0.816773	0.806719
0.045	0.778108	0.79587	0.782543	0.804093	0.787627	0.777452	0.8206	0.815129
0.046	0.777751	0.813964	0.783637	0.802104	0.783831	0.769773	0.822635	0.806961
0.047	0.777132	0.806924	0.790419	0.802996	0.772236	0.770026	0.820518	0.806888
0.048	0.779238	0.814132	0.789246	0.794259	0.761192	0.773452	0.82066	0.796003
0.049	0.772449	0.808317	0.7913	0.801568	0.760802	0.769094	0.825351	0.798479
0.05	0.771925	0.816346	0.784209	0.791754	0.785647	0.779662	0.819667	0.781801
0.051	0.775474	0.811622	0.784214	0.788867	0.756503	0.791892	0.821178	0.782318
0.052	0.766145	0.774613	0.794763	0.78531	0.782914	0.779326	0.817836	0.778501
0.053	0.767935	0.801185	0.792517	0.781143	0.751971	0.777554	0.817627	0.771619
0.054	0.7541	0.782999	0.7836	0.793138	0.774556	0.768761	0.807756	0.776877
0.055	0.763045	0.779731	0.77292	0.767734	0.725738	0.76656	0.810326	0.748682
0.056	0.763456	0.785778	0.764363	0.774169	0.701157	0.759874	0.811916	0.756718
0.057	0.750904	0.76206	0.771776	0.779297	0.741788	0.760834	0.79032	0.736901
0.058	0.726305	0.74282	0.770297	0.757936	0.694182	0.733714	0.766625	0.717412
0.059	0.728299	0.752177	0.750217	0.758247	0.688383	0.735728	0.76703	0.727945
0.06	0.724591	0.756355	0.748492	0.756373	0.693739	0.731093	0.763946	0.730063
0.061	0.714906	0.733797	0.690521	0.716383	0.653878	0.679704	0.742868	0.689382
0.062	0.730258	0.742756	0.694486	0.719942	0.669409	0.677025	0.745476	0.729472
0.063	0.742305	0.752099	0.732452	0.753294	0.738051	0.693479	0.749615	0.763887
0.064	0.718712	0.730588	0.695247	0.714462	0.69809	0.650049	0.723218	0.730493
0.065	0.718712	0.730588	0.718457	0.714462	0.69809	0.673669	0.723218	0.731565
0.066	0.718006	0.729724	0.735253	0.73641	0.692093	0.674112	0.719975	0.740287

Supplementary Table S1. Manual Optimized Classifier Performances on Validation Set.

	optimal threshold	ROC AUC	PR AUC	Accuracy	Sensitivity	Specificity
SVM	0.038	0.81	0.79	0.72(0.62-0.80)	0.63(0.48-0.76)	0.79(0.66-0.88)
MLP	0.050	0.83	0.83	0.79(0.70-0.86)	0.61(0.46-0.74)	0.93(0.83-0.98)
RF	0.013	0.77	0.75	0.73(0.63-0.81)	0.63(0.48-0.76)	0.80(0.68-0.89)
xgboost	0.034	0.82	0.78	0.75(0.65-0.83)	0.66(0.51-0.79)	0.82(0.70-0.90)
Adaboost	0.036	0.80	0.70	0.72(0.62-0.80)	0.61(0.46-0.74)	0.80(0.68-0.89)
ET	0.027	0.79	0.72	0.73(0.63-0.81)	0.66(0.51-0.79)	0.79(0.66-0.88)
LR	0.031	0.89	0.89	0.84(0.75-0.90)	0.76(0.61-0.87)	0.89(0.78-0.95)
GB	0.028	0.77	0.75	0.73(0.63-0.81)	0.63(0.48-0.76)	0.80(0.68-0.89)

Supplementary Table S2. Validation Set Pipeline Performances on TPOT Light Analysis.

	AUC	pipeline
1	0.787	make_pipeline(Binarizer(threshold=1.0), LogisticRegression(C=0.5, dual=False, penalty="l2"))
2	0.747	make_pipeline(MinMaxScaler(), KNeighborsClassifier(n_neighbors=61, p=2, weights="distance"))
3	0.745	make_pipeline(StackingEstimator(estimator=LogisticRegression(C=0.01, dual=False, penalty="l2")), StandardScaler(), Binarizer(threshold=0.75), KNeighborsClassifier(n_neighbors=48, p=2, weights="distance"))
4	0.751	make_pipeline(MaxAbsScaler(), MaxAbsScaler(), KNeighborsClassifier(n_neighbors=59, p=2, weights="uniform"))
5	0.770	make_pipeline(MinMaxScaler(), KNeighborsClassifier(n_neighbors=18, p=2, weights="distance"))
6	0.726	make_pipeline(SelectFwe(score_func=f_classif, alpha=0.014), StandardScaler(), LogisticRegression(C=25.0, dual=False, penalty="l2"))
7	0.773	make_pipeline(make_union(FeatureAgglomeration(affinity="euclidean", linkage="average"), SelectFwe(score_func=f_classif, alpha=0.048)), DecisionTreeClassifier(criterion="entropy", max_depth=6, min_samples_leaf=20, min_samples_split=17))
8	0.761	make_pipeline(Binarizer(threshold=0.05), KNeighborsClassifier(n_neighbors=76, p=1, weights="uniform"))
9	0.777	make_pipeline(MinMaxScaler(), KNeighborsClassifier(n_neighbors=26, p=2, weights="uniform"))
10	**0.801**	**make_pipeline(SelectFwe(score_func=f_classif, alpha=0.014), StandardScaler(), LogisticRegression(C=25.0, dual=False, penalty="l2"))**

Supplementary Table S3. Validation Set Pipeline Performances on TPOT Analysis

	AUC	pipeline
1	0.814	ExtraTreesClassifier(input_matrix, bootstrap=True, criterion=entropy, max_features=1.0, min_samples_leaf=15, min_samples_split=8, n_estimators=100)
2	0.800	ExtraTreesClassifier(input_matrix, bootstrap=True, criterion=entropy, max_features=0.35000000000000003, min_samples_leaf=5, min_samples_split=6, n_estimators=100)
3	0.811	make_pipeline(VarianceThreshold(threshold=0.001),OneHotEncoder (minimum_fraction=0.1, sparse=False, threshold=10), GradientBoostingClassifier(learning_rate=0.01, max_depth=8, max_features=0.1, min_samples_leaf=17, min_samples_split=9, n_estimators=100, subsample=0.7500000000000001))
4	0.814	make_pipeline(StackingEstimator(estimator=GradientBoostingClassifier(learning_rate=0.01, max_depth=10, max_features=0.15000000000000002, min_samples_leaf=7, min_samples_split=3, n_estimators=100, subsample=0.3)), StackingEstimator(estimator=LinearSVC(C=0.0001, dual=True, loss="hinge", penalty="l2", tol=0.001)), ExtraTreesClassifier(bootstrap=False, criterion="entropy", max_features=0.25, min_samples_leaf=8, min_samples_split=11, n_estimators=100))
5	0.818	RandomForestClassifier(input_matrix, bootstrap=True, criterion=entropy, max_features=0.05, min_samples_leaf=11, min_samples_split=2, n_estimators=100)
6	0.780	make_pipeline(StackingEstimator(estimator=ExtraTreesClassifier(bootstrap=True, criterion="entropy", max_features=0.6000000000000001, min_samples_leaf=13, min_samples_split=3, n_estimators=100)), RFE(estimator=ExtraTreesClassifier(criterion="gini", max_features=0.4, n_estimators=100), step=0.9000000000000001), StackingEstimator(estimator=GaussianNB()), XGBClassifier(learning_rate=0.01, max_depth=6, min_child_weight=6, n_estimators=100, n_jobs=1, subsample=0.6500000000000001, verbosity=0))
7	0.793	make_pipeline(StackingEstimator(estimator=SGDClassifier(alpha=0.01, eta0=1.0, fit_intercept=False, l1_ratio=0.25, learning_rate="constant", loss="hinge", penalty="elasticnet", power_t=10.0)), RandomForestClassifier(bootstrap=True, criterion="gini", max_features=0.1, min_samples_leaf=5, min_samples_split=20, n_estimators=100))

8	0.805	make_pipeline(StackingEstimator(estimator=GradientBoostingClassifier(learning_rate=0.1, max_depth=5, max_features=0.6500000000000001, min_samples_leaf=19, min_samples_split=4, n_estimators=100, subsample=0.8)), ExtraTreesClassifier(bootstrap=True, criterion="entropy", max_features=0.35000000000000003, min_samples_leaf=16, min_samples_split=10, n_estimators=100))
**9**	**0.825**	**make_pipeline(VarianceThreshold(threshold=0.0001), RandomForestClassifier(bootstrap=True, criterion="entropy", max_features=0.3, min_samples_leaf=17, min_samples_split=18, n_estimators=100))**
10	0.824	make_pipeline(OneHotEncoder(minimum_fraction=0.1, sparse=False, threshold=10), RandomForestClassifier(bootstrap=True, criterion="entropy", max_features=0.7000000000000001, min_samples_leaf=14, min_samples_split=10, n_estimators=100))

Supplementary Table S4. Validation Set Performances of TPOT.

	ROC AUC	PR AUC	Accuracy	Sensitivity	Specificity
TPOT Light	0.80	0.82	0.80(0.71-0.87)	0.54(0.39-0.68)	1.00(0.92-1.00)
TPOT	0.83	0.81	0.77(0.68-0.84)	0.76(0.61-0.87)	0.79(0.66-0.88)
